# Effect of Nano-Al_2_O_3_ on the Toxicity and Oxidative Stress of Copper towards *Scenedesmus obliquus*

**DOI:** 10.3390/ijerph13060575

**Published:** 2016-06-09

**Authors:** Xiaomin Li, Suyang Zhou, Wenhong Fan

**Affiliations:** Environment Science and Engineering, School of Space and Environment, Beihang University, Beijing 100191, China; xiaominli@buaa.edu.cn (X.L.); zhousuyangr@163.com (S.Z.)

**Keywords:** nano-Al_2_O_3_, copper, *Scenedesmus obliquus*, toxicity

## Abstract

Nano-Al_2_O_3_ has been widely used in various industries; unfortunately, it can be released into the aquatic environment. Although nano-Al_2_O_3_ is believed to be of low toxicity, it can interact with other pollutants in water, such as heavy metals. However, the interactions between nano-Al_2_O_3_ and heavy metals as well as the effect of nano-Al_2_O_3_ on the toxicity of the metals have been rarely investigated. The current study investigated copper toxicity in the presence of nano-Al_2_O_3_ towards *Scenedesmus obliquus*. Superoxide dismutase activity and concentration of glutathione and malondialdehyde in cells were determined in order to quantify oxidative stress in this study. Results showed that the presence of nano-Al_2_O_3_ reduced the toxicity of Cu towards *S. obliquus*. The existence of nano-Al_2_O_3_ decreased the growth inhibition of *S. obliquus*. The accumulation of copper and the level of oxidative stress in algae were reduced in the presence of nano-Al_2_O_3_. Furthermore, lower copper accumulation was the main factor that mitigated copper toxicity with the addition of nano-Al_2_O_3_. The decreased copper uptake could be attributed to the adsorption of copper onto nanoparticles and the subsequent decrease of available copper in water.

## 1. Introduction

Nano-Al_2_O_3_ is commonly used as catalyst support, dispersing agent, coating, and abrasive in the fields of microelectronics, ceramics, and biomedicine. Researchers have also attempted to utilize nano-Al_2_O_3_ as a coagulant in wastewater treatment as well as a component in the removal of organic matter [1]. As these applications release nano-Al_2_O_3_ into the environment, especially aquatic environments, they may have an impact on aquatic organisms in these environments [2,3]. The toxicity of nano-Al_2_O_3_ has been investigated with different organisms, such as bacteria, zooplankton, and phytoplankton [4,5,6]. The 72 h-EC_50_ values of nano-Al_2_O_3_ to *Scenedesmus* sp. and *Chlorella* sp. are 39.35 mg/L and 45.4 mg/L, respectively [7]. The 24 h-LC_50_ value of nano-Al_2_O_3_ to *Caenorhabditis elegans* is 153 mg/L [4], which indicates low toxicity at environmentally relevant concentrations. Considering that the background concentration of nano-Al_2_O_3_ might be in ng/L to µg/L in the aquatic environment [8,9,10], the toxicity of nano-Al_2_O_3_ itself is deemed insignificant. 

However, when nano-Al_2_O_3_ is released into an aquatic environment, it could interact with the other water pollutants, such as heavy metals. This interaction may produce an unpredictable impact on the ecological effects of the metals. Much concern has been raised in recent years concerning the synergistic toxic effect of nanoparticles and metals. Nanoparticles have large specific surface areas; thus, they could easily adsorb metals on their surface. Nano-Al_2_O_3_ is considered a promising sorbent for heavy metals in water [11]; it has great potential as an adsorbent to remove heavy metals, such as Pb (II), Cd (II), Cr (VI), and Cu (II), from wastewater [12,13]. 

Despite the possible co-existence of nano-Al_2_O_3_ and metals in aquatic environments, the influence of nano-Al_2_O_3_ on the toxicity of heavy metals has been rarely evaluated. Most of the related studies have focused on nano-TiO_2_ [14,15,16,17,18,19,20,21], whose effects on heavy metal toxicity vary with the type of heavy metal and characteristics/concentration of nanoparticles and model organisms. For example, the toxicity and bioavailability of Cd^2+^ and Cr(VI) to *Chlamydomonas reinhardtii* decreases in the presence of TiO_2_ [14], whereas the low concentration of nano-TiO_2_ increases the toxicity of Zn^2+^ significantly [15]. When nano-TiO_2_ concentration increases, the toxicity of the Zn^2+^/TiO_2_ system decreases [15]. Nano-TiO_2_ of different sizes exerts different effects on Cd toxicity in *Pseudokirchneriella subcapitata* [16]; it can also serve as a carrier of Cd in a protozoan *Tetrahymena thermophila* and increased Cd bioaccumulation in the protozoan [17]. The presence of nano-TiO_2_ increases Ag toxicity for juveniles, while the toxicity of As and Cu towards *Daphnia magna* is reduced [18,19]. Another study reported that Cu-induced toxicity towards the benthic amphipod *Gammarus fossarum* was significantly reduced in the presence of nano-TiO_2_ [20]. The adsorption of metals onto the surface of nanoparticles is one of the reasons to which the changes in metal toxicity are attributed. However, few results on the synergistic toxic effect of nano-Al_2_O_3_ and heavy metals have been reported. Nano-Al_2_O_3_ did not show a significant effect on Cr(VI) toxicity towards the growth of *Scenedesmus* sp. [21], although it can significantly increase the toxicity of As(V) to *Ceriodaphnia dubia* [22]. 

Copper is an essential element that facilitates various biochemical processes, such as photosynthetic and respiratory electron transport [23]. However, copper can be toxic at high concentrations [24]. Studies have indicated that excessive amounts of copper inhibit the growth and photosynthesis of microalgae [25,26], change the antioxidant defense mechanism, and cause lipid peroxidation [27,28]. 

Copper is commonly detected in waters and is one of the priority pollutants identified by the US EPA [24,29]. Pollution incidents caused by copper mining or electroplating wastewater often occur [30,31]. Once nano-Al_2_O_3_ is released into an aquatic environment, it may inevitably co-exist with copper. In the current study, the effect of nano-Al_2_O_3_ on the behavior and toxicity of copper to *Scenedesmus obliquus* was investigated. The growth inhibition of *S. obliquus* and the change in its antioxidant defense were investigated.

## 2. Materials and Methods

### 2.1. Materials

*Scenedesmus obliquus* was obtained from the Institute of Hydrobiology, the Chinese Academy of Sciences, Wuhan, China. Algae cells were cultured in a BG11 medium at 23 ± 1 °C with light illumination of 7300 lux in a 12:12 light–dark cycle. The samples were maintained under static conditions and shaken four times a day so that they would not stick to the container wall. After arriving at the mid-exponential growth phase, the cells were utilized for a toxicity test.

Commercial nano-Al_2_O_3_ particles were obtained from Beijing Boyu High-tech New Material Co., Ltd., Beijing, China. The particles had a specific surface area of 110.77 m^2^/g. The mean particle diameter was 20 nm, and the crystalline phase was γ-phase. 

### 2.2. Test Medium

A modified BG11 medium was utilized as the test medium in the toxicity experiments. The BG11 medium contains a chelating agent (EDTA-Na_2_) and metals (Mn^2+^, Co^2+^), which could affect the state and behavior of copper and lead to the formation of precipitates. The medium was modified to eliminate the metals and EDTA-Na_2_ according to the procedure of Yang *et al.* [32]. The ionic strength of modified BG11 medium was 0.02 mol/L. The composition of the modified medium is shown in Table 1. 

### 2.3. Preparation of Exposure Solution and Its Characterization

Nano-Al_2_O_3_ was suspended in the modified BG11 medium by sonication in a water bath for 30 min. Cu(NO_3_)_2_ solution was then added to the dispersed nano-Al_2_O_3_ solution to create a nano-Al_2_O_3_-Cu suspension as the exposure solution. The containers were placed on a shaker (200 rpm) at 20 ± 1 °C and mixed for 1 h.

The state of the particles was observed by using a transmission electron microscope (TEM) (JEOL, JEM-2100F, Tokyo, Japan). Nano-Al_2_O_3_ was suspended in the modified BG11 medium through a 30 min sonication in a water bath (1.0 mg/L). Droplets of the solution were dripped onto Formvar-coated copper grids (Electron Microscopy Sciences, Fort Washington, PA, USA). The samples were then allowed to dry before being placed in the TEM for imaging (100 kV). Next, hydrodynamic particle size and zeta potential were monitored with Zetasizer Nano ZS at different time periods (0, 24, 48, and 72 h). The analysis was repeated three times, and the average of all values was calculated.

### 2.4. Adsorption of Copper onto Nano-Al_2_O_3_

The adsorption of copper onto nano-Al_2_O_3_ was determined by adding copper into the nano-Al_2_O_3_ suspension at copper concentrations of 0, 0.05, and 0.5 mg/L. The adsorption experiments lasted for 6 h, and samplings were conducted after 10, 20, 30, 40, 50, 60, 180, and 360 min. At each time point, 2 mL of the mixture was taken out and centrifuged for 10 min at 12,000 rpm (Himac CF 16RX, Hitachi, Tokyo, Japan) [33]. The concentration of copper in the supernatant was determined through inductively coupled plasma mass spectrometry (ICP-MS) (VG PQ2 TURBO, Cheshire, UK). The adsorption percentages of the copper were calculated as follows [34]:

(1) $$\%\;Adsorption=\frac{\left(C_{0}-C_{e}\right)}{C_{e}}\times 100$$

where C_0_ and C_e_ served as the initial and equilibrium concentrations of copper (mg/L), respectively.

### 2.5. Toxicity Test

*S. obliquus* cells were harvested at their exponential phase from the BG11 medium through centrifugation (4500 rpm, 15 min, 0 °C) and washed thoroughly with the modified BG11 medium. The cells were then re-suspended in an exposure solution. The initial cell count was approximately 4×10^5^ cells/mL for each treatment. 

A toxicity test was conducted for 72 h in a climate chamber at 23 ± 1 °C with light illumination of 7300 lux in a 12 h:12 h light–dark cycle. The samples were maintained under static condition and shaken four times a day to prevent the cells from sticking to the container wall. The exposure solution contained different concentrations of dissolved copper with or without nano-Al_2_O_3_ particles. The concentrations of copper and nano-Al_2_O_3_ in the exposure solutions are shown in Table 2. Each treatment was implemented three times.

The samples were taken out during the experiments at four time points (0, 24, 48, and 72 h) to measure the algal growth with a UV-Vis spectrophotometer (650 nm) [35]. Growth inhibition was calculated for each treatment as follows [36]:

(2) $${\text{\% I}}_{y}=\frac{\left(Y_{C}-Y_{T}\right)}{Y_{C}}\times 100$$

where

% I_y_: growth inhibition;
Y_C_: mean value of cell number in the control group; and
Y_T_: value of cell number for the treatment replicate.

After 72 h, *S. obliquus* cells were collected through centrifugation (4500 rpm, 15 min, 0 °C) and divided into two subsamples. One was suspended in a phosphate buffer (0.05 mol/L, pH 7.0), homogenized in an ice bath by ultrasonication, and then centrifuged for 10 min at 12,000 rpm. The supernatant was analyzed for superoxide dismutase (SOD) activity and concentrations of reduced glutathione (GSH) and malondialdehyde (MDA) with commercial kits (Nanjing Jiancheng Bioengineering Institute, Nanjing, China) according to the manufacturer’s protocol. SOD activity was measured based on its inhibition of NADPH oxidation by molecular oxygen in the presence of EDTA, manganese chloride, and 2-mercapto-ethanol, and then expressed as U/mg protein [37]. GSH assay was determined with 5,5-dithiobis-2-nitrobenzoic acid (DTNB) colorimetric method, and the concentration was expressed as g GSH/L [38]. MDA concentration was determined with thiobarbituric acid colorimetric method and expressed as nmol MDA/mg protein [39].

Another sub-sample was washed with 10 mM EDTA-Na_2_ to remove the Cu^2+^ absorbed onto the cell wall, and then digested with 68% HNO_3_ at 110 °C until the solution became completely transparent. The digestion tube was then washed with 2% nitric acid, and the entire solution was transferred to a volumetric flask. The volume of the solution was adjusted to 10 mL with 2% HNO_3_ [40]. Copper concentration in the solution was analyzed through ICP-MS (VG PQ2 TURBO, Cheshire, UK). EDTA-Na_2_ was able to remove the Cu^2+^ that was weakly adsorbed on the algal cells. However, this was not the case when both nanoparticles and algal cells were present simultaneously, and the accumulation of copper in the cells would be overestimated [32]. To further eliminate the interference of surface-adsorbed copper, copper accumulation was quantified through the subtraction of 15 min copper bioaccumulation after the beginning of exposure from that measured with 72 h exposure [32]. 

### 2.6. Statistical Analysis

All data are presented as mean values ± standard deviation. Statistical analysis was used to compare significant differences through the *t*-test. The correlation of parameters was determined through a Pearson correlation coefficient analysis. *p*-values of less than 0.05 were considered statistically significant. All data were analyzed with SPSS software (IBM SPSS Statistics version 19.0, Chicago, IL, USA) and Origin software (OriginLab Origin 9.0, Northampton, MA, USA). 

## 3. Results and Discussion

### 3.1. Interaction of Copper and Nano-Al_2_O_3_

To determine the effect of copper on the stability of nano-Al_2_O_3_, the mean hydrodynamic diameter (MHD) and zeta potential of the exposure solution were measured. The original diameter of the nano-Al_2_O_3_ particles was approximately 20 nm. When suspended in the exposure solution, MHD increased rapidly over time and reached 1700–1900 nm after 72 h (Table 3). However, the particles did not show increasing aggregation at different copper concentrations, suggesting that copper could not affect the aggregation of nano-Al_2_O_3_. Similar results were reported that nano-Al_2_O_3_ (original particle size <50 nm) aggregated to 1204.4 ± 140.5 nm after 72 h and was unaffected by the existence of Cr(VI) [21]. 

**Figure 1:**
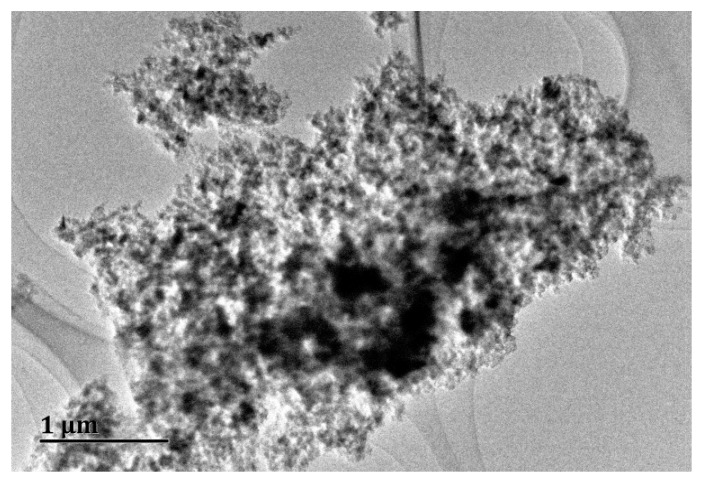
Transmission electron mocroscopy (TEM) image of nano-Al_2_O_3_ in the exposure solution. Nano-Al_2_O_3_ was agglomerated to large particles (about 1000–3000 nm).

No significant changes in zeta potential were found at different concentrations of copper (Table 3). The zeta potential of the particles was approximately −20 mV in the solution. According to a previous study, when the zeta potential is between −30 mV and 30 mV, the suspension is unlikely to be stable and is prone to aggregation [41]. This is the reason behind the obvious agglomeration of nano-Al_2_O_3_, as observed from the TEM images (Figure 1). The mean hydrodynamic diameter of the particles suspended in the exposure solution increased to approximately 1000–3000 nm as shown in Figure 1. The particle sizes were beyond their original nanoscale size in the exposure solution, but they were still different from their bulk counterparts [15,42].

**Figure 2:**
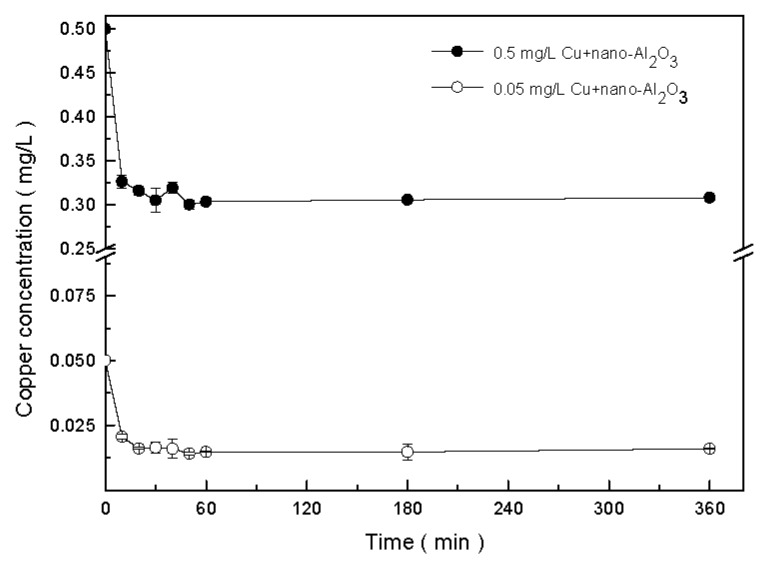
Adsorption of copper onto nano-Al_2_O_3_ in the modified BG11 medium over 6 h. Data are mean ± SD (*n* = 2).

Next, the change in the concentration of free copper in the solution after contact with nano-Al_2_O_3_ was measured, as shown in Figure 2. From the original concentrations of 0.05 and 0.5 mg/L, the concentrations of free copper decreased rapidly within the first 60 min and then slowly decreased to 0.016 and 0.308 mg/L, respectively. The adsorption capacity of copper on nano-Al_2_O_3_ could reach 100% at high nano-Al_2_O_3_ doses (2 g/L) [43]. In the current experiments, the copper adsorption rates were approximately 38% and 68% at copper concentrations of 0.5 and 0.05 mg/L, respectively.

The adsorption of copper onto nano-Al_2_O_3_ could be attributed to the complex surface interaction between the functional group ≡AlOH and metallic ion, which is given as follows [44]:

≡AlOH + M^2+^ ↔ ≡AlO-M^+^ + H^+^ (M = Cu) (3)

Therefore, pH has an impact on the adsorption capacity of the metal [45]. With an increase in pH, many adsorption sites (≡AlO-) are present, leading to high adsorption. Sun *et al.* found that the adsorption of Cu(II) on nano-Al_2_O_3_ reached its maximum at pH 7.5 [46]. In the current study, the pH of the modified BG11 medium was 7.4 [47], indicating that an amount of copper was adsorbed on nano-Al_2_O_3_ as expected. The ionic strength of solution (0.02 mol/L) also might affect the aggregation of nanoparticles and the adsorption of metal ions on particles [48,49,50,51]. 

### 3.2. Influence of Nano-Al_2_O_3_ on the Toxicity of Copper

Copper is an essential micronutrient for organisms, but it could be toxic depending on its concentration. The growth inhibition of *S. obliquus* by Cu in the absence or presence of nano-Al_2_O_3_ is shown in Table 4. In the copper-exposed treatments, the cell number decreased gradually with the increase in copper concentration. The exposure of *S. obliquus* to copper resulted in significant differences in cell number between the control and experimental samples (*p* < 0.05). High initial copper concentration led to high growth inhibition. In addition, growth inhibition was 33.33% when copper concentration was 0.5 mg/L.

The EC_50_ of nano-Al_2_O_3_ to algal growth is more than 30 mg/L [7,52]. The toxicity of nano-Al_2_O_3_ itself at 1.0 mg/L could be neglected. No significant difference in cell growth was observed between the control and experimental samples when algae was exposed to 1.0 mg/L nano-Al_2_O_3_ (*p* > 0.5, Effect Size (ES) = 0.93). However, in the copper/Al_2_O_3_ system, significant differences between the control and experimental samples (*p* < 0.05, ES > 0.5) were observed. Growth inhibition decreased compared with that of copper exposure treatments. This observation indicated that the presence of nano-Al_2_O_3_ dramatically mitigated growth inhibition, especially at high copper concentrations (0.2 and 0.5 mg/L) (*p* < 0.05, ES = 0.91 (0.2 mg/L), ES = 13.17 (0.5 mg/L)). Growth inhibition was 33.33% at a copper concentration of 0.5 mg/L in the copper-exposed treatment and only 23.29% in the copper/nano-Al_2_O_3_-exposed treatment. Growth inhibition decreased by about 30% compared with that of the copper-exposed treatments. 

### 3.3. Accumulation of Copper in S. obliquus

Metal uptake is one of the major mechanisms in the toxicity of heavy metals towards organisms [24]. Levels of copper accumulation in *S. obliquus* after exposure to copper with and without 1.0 mg/L nano-Al_2_O_3_ are illustrated in Table 4. As can be seen, copper accumulation increased with copper concentration. In copper-exposed treatments, accumulation was between 35.47 ng/mg (dry wt) and 596.04 ng/mg (dry wt). In copper/nano-Al_2_O_3_-exposed treatments, the values were 41.43 ng/mg (dry wt)–458.21 ng/mg (dry wt). The difference in the accumulation between both treatments became significant with the increase in copper concentration. In particular, the accumulation with copper/nano-Al_2_O_3_ treatments was 23.1% lower than that with copper treatments when the concentration of copper was 0.5 mg/L. Hence, the presence of nano-Al_2_O_3_ dramatically reduced the accumulation of copper in *S. obliquus*.

### 3.4. Oxidative Stress and Antioxidant Defense

Aside from the accumulation of copper in cells, metal pollution also causes oxidative damage [53]. Algal tolerance for heavy metal pollution partly depends on its defense responses to prevent oxidative damage [54]. The change in the antioxidant defense system can be measured with SOD and GSH as biomarkers. 

**Figure 3:**
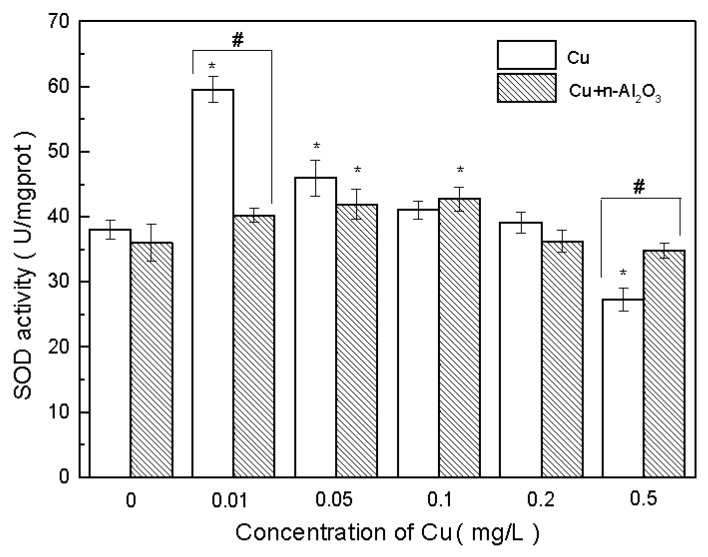
Superoxide dismutase (SOD) activities in *S. obliquus* exposed to copper and copper/nano-Al_2_O_3_ systems at different concentrations of copper after 72 h. Data are mean ± SD (*n* = 3). Asterisks indicate the significance of differences (*p* < 0.05) between the control and the exposed treatments. Pound signs indicate the significance of differences (*p* < 0.05) between copper system and copper/nano-Al_2_O_3_ system at the same concentration of copper.

SOD is an enzymatic antioxidant that can scavenge reactive oxygen species (ROS) generated by heavy metal stress. The change in SOD activity during the experiments is shown in Figure 3. As can be seen in the figure, exposure of *S. obliquus* to 1.0 mg/L nano-Al_2_O_3_ did not lead to changes in SOD activity (*p* > 0.5, ES = 1.08). However, with the increase in copper concentration, SOD activity increased initially at a low concentration and then decreased either with or without nano-Al_2_O_3_. 

However, with copper/nano-Al_2_O_3_ treatments, the change in SOD activity was retarded. SOD activity in the presence of nano-Al_2_O_3_ at a low copper concentration (0.01 mg/L) was significantly lower than that with only copper (*p* < 0.05, ES = 13.31). At a copper concentration of 0.5 mg/L, SOD activity in the copper/nano-Al_2_O_3_ system was higher than that in the copper system (*p* < 0.05, ES = 4.57). 

*S. obliquus* exhibits hormesis toward copper, *i.e.*, low-dose stimulation and high-dose inhibition [55]. When SOD activity is stimulated, its ability to eliminate free radicals is improved at a low concentration of the toxicant, thereby protecting algae from oxidative damage [56]. At a high concentration, this ability exceeds its effect and results in the destruction of the anti-oxidation enzyme system and the inhibition of SOD activity. This was the reason why SOD activity in the copper system was lower than that of the copper/nano-Al_2_O_3_ system at a copper concentration of 0.5 mg/L in the current study. However, under the presence of nano-Al_2_O_3_, this hormesis effect of copper was retarded. The decrease in SOD in this study occurred at >0.1 mg/L of copper under the presence of nano-Al_2_O_3_, but at >0.01 mg/L without nano-Al_2_O_3_. 

**Figure 4:**
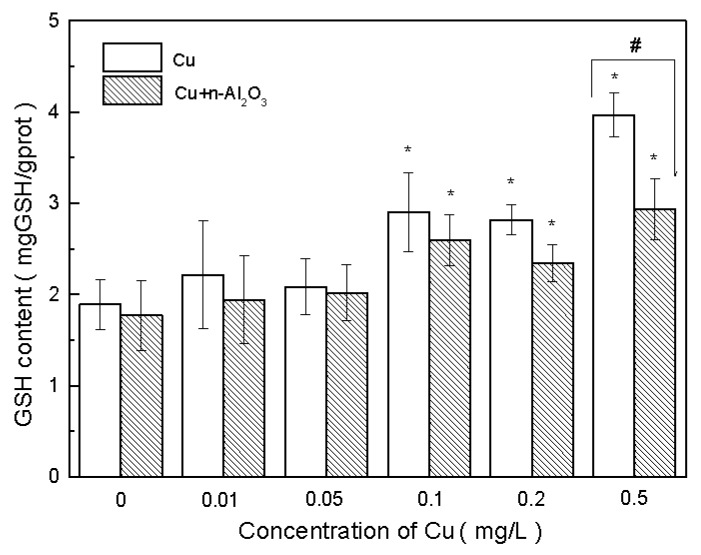
Reduced glutathione (GSH) concentrations in *S. obliquus* exposed to copper and copper/nano-Al_2_O_3_ systems at different concentrations of copper after 72 h. Data are mean ± SD (*n* = 3). Asterisks indicate the significance of differences (*p* < 0.05) between the control and the exposed treatments. Pound signs indicate the significance of differences (*p* < 0.05) between the copper system and the copper/nano-Al_2_O_3_ system at the same concentration of copper.

GSH is another antioxidant and major free radical remover. Algae can respond to heavy metal stress by increasing the GSH concentration. The influence of GSH concentration by Cu and nano-Al_2_O_3_ is shown in Figure 4. No significant difference in GSH concentration was observed under the exposure of 1.0 mg/L nano-Al_2_O_3_ (*p* > 0.5, ES = 0.21). However, with the increase in copper concentration, the GSH concentrations increased gradually in both copper and copper/nano-Al_2_O_3_ systems. 

The GSH concentrations of the copper treatments were higher than those of copper/nano-Al_2_O_3_ treatments under the same copper concentrations. When the copper concentration was 0.5 mg/L, the GSH concentration in the copper treatments was significantly higher than that in copper/nano-Al_2_O_3_ treatments (*p* < 0.05, ES = 4.40). Therefore, nano-Al_2_O_3_ also decreased the demand of GSH to remove free radicals. Furthermore, nano-Al_2_O_3_ alleviated the oxidative stress of Cu towards *S. obliquus* with regard to the functions of SOD and GSH. 

**Figure 5:**
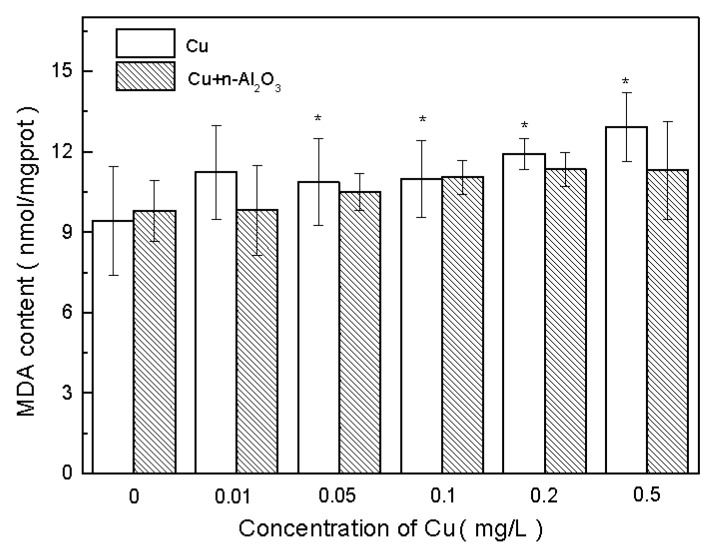
Malondialdehyde (MDA) concentrations in *S. obliquus* exposed to copper and copper/nano-Al_2_O_3_ systems at different concentrations of copper after 72 h. Data are mean ± SD (*n* = 3). Asterisks indicate the significance of differences (*p* < 0.05) between the control and the exposed treatments. Pound signs indicate the significance of differences (*p* < 0.05) between the copper system and the copper/nano-Al_2_O_3_ system at the same concentration of copper.

MDA is another biomarker of free radical damage—one that serves as an indicator of cell membrane damage [57]. The change in MDA concentration during exposure is shown in Figure 5. As can be seen, an amount of 1.0 mg/L nano-Al_2_O_3_ did not exert a significant influence on MDA concentration (*p* > 0.5, ES = 0.4). Moreover, no obvious differences were observed at different concentrations compared with the control in copper/nano-Al_2_O_3_-exposed treatments (*p* > 0.05, ES > 0.8). However, with the increase in copper concentration, MDA concentration also increased in both exposure systems. 

Moreover, no large differences in MDA were observed between copper and copper/nano-Al_2_O_3_ exposure (*p* > 0.05, ES > 0.8). Therefore, the presence of nano-Al_2_O_3_ did not cause changes in cell membrane damage. This condition might be related to the low copper concentrations in the experiments, which were insufficient to generate a large amount of MDA. Microalgae have been reported to produce high MDA at 26.5 mg/L copper [58], which is much higher than the concentration recorded in the current study. 

### 3.5. Mechanism of Reduced Copper Toxicity by Nano-Al_2_O_3_

In the current study, the concentration of nano-Al_2_O_3_ was only 1.0 mg/L. No significant changes in growth inhibition, SOD activity, and GSH and MDA concentrations were observed under the exposure of 1.0 mg/L nano-Al_2_O_3_. Therefore, the toxicity of nano-Al_2_O_3_ itself to algae can be neglected. However, its existence changed the toxicity and concentration of bioindicators in *S. obliquus*. Thus, to determine the mechanism of nano-Al_2_O_3_ effect on copper toxicity, the correlations among growth inhibition and copper accumulation, SOD activity, and GSH and MDA concentrations in both systems were investigated. 

**Figure 6:**
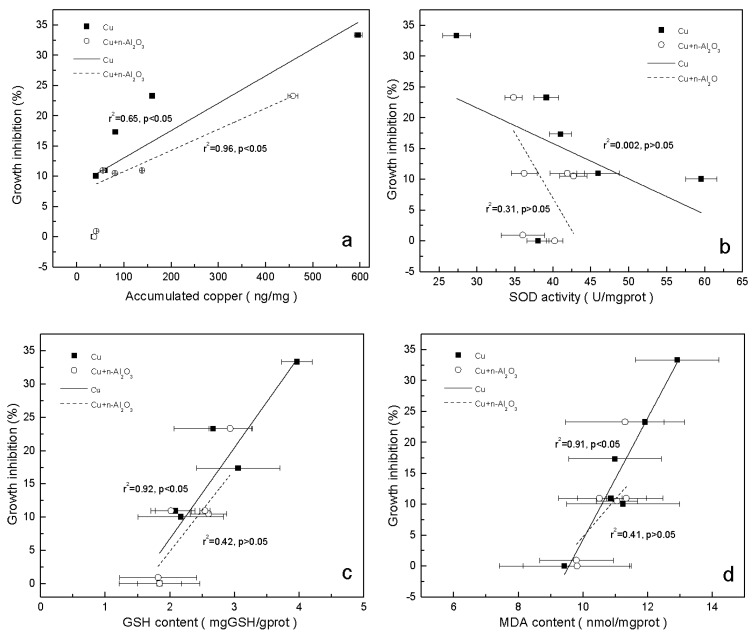
Correlation between growth inhibition and (**a**) accumulated Cu; (**b**) SOD activity; (**c**) GSH concentration and (**d**) MDA concentration (mean ± SD) in *S. obliquus* exposed to copper and copper/nano-Al_2_O_3_ systems at different concentrations of copper after 72 h.

Growth inhibition had a definite positive correlation with Cu accumulation in both systems (*p* < 0.05) (Figure 6a), suggesting that the mitigation of copper toxicity by nano-Al_2_O_3_ could have resulted from the decrease in copper accumulation in *S. obliquus*. Results also showed that the growth inhibition of the algae and accumulated Cu cannot be fitted to the same dose-response curve with and without the addition of nano-Al_2_O_3_. With similar Cu accumulation, the growth inhibition with nano-Al_2_O_3_ was lower than that without nano-Al_2_O_3_, indicating that nano-Al_2_O_3_ showed a beneficial effect on algae growth apart from the impact on copper accumulation. The treatments exposed to 1.0 mg/L nano-Al_2_O_3_ also showed the promotion of algal growth in the first two days (Table 4) compared with the control. Although an inhibition effect was found at 72 h, it was quite low (0.91%). Other studies also found that nano-Al_2_O_3_ and nano-Al could promote growth in algae and roots of plants in some cases [59,60].

According to Figure 6b, SOD activity was not correlated with growth inhibition in both systems (*p* > 0.05). The increase in copper toxicity might not be necessarily a result of the change in SOD activity. This might be explained by the hormesis effect of SOD activity from copper. Although the existence of nano-Al_2_O_3_ weakened this effect, SOD activity still increased initially and then decreased with the increase in copper concentration. GSH and MDA concentration had a positive correlation with growth inhibition in the copper-exposed treatments (*p* < 0.05) but was not correlated in copper/nano-Al_2_O_3_-exposed treatments (*p* > 0.05) (Figure 6c,d). Although the correlation in the copper/nano-Al_2_O_3_ system was insignificant, the GSH concentration also showed an increasing trend. GSH plays a crucial role in algal detoxification of heavy metals by forming stable metal–glutathione complexes [61]. A high GSH level has also been reported in Cu-tolerant and Cd-tolerant algae [62]. Meanwhile, the presence of nano-Al_2_O_3_ reduced copper toxicity through the mitigation of membrane damage. Sabatini *et al.* reported that the increase in MDA content has a linear relation with the increase in internal copper content [58], which is in accordance with the results shown in Figure 6d. 

The exposure of Cu resulted in the accumulation of Cu in *S. obliquus* and the toxicity of Cu to algae can be partly related to the generation of free radicals and subsequent oxidative stress [63,64]. The results showed that when Cu concentration was low, SOD activity was enhanced while GSH concentration decreased. Hence, SOD served as the main free radical remover in this situation. When Cu concentration increased, SOD activity was inhibited while GSH concentration increased. GSH was the main free radical scavenger in this situation. However, when the stress of copper exceeded the self-protection capacity of cells, the excessive free radicals could not be removed in time [65]. This was the reason behind the occurrence of membrane lipid peroxidation, as indicated by the MDA level. However, the MDA concentration was low, indicating low membrane lipid peroxidation. Hence, the existence of nano-Al_2_O_3_ might not change the mechanism of copper toxicity; however, it decreased copper accumulation in algae and further decreased the toxicity of copper. 

The reason why copper accumulation decreased with the existence of nano-Al_2_O_3_ was investigated. As stated above, a large amount of copper was adsorbed onto the surface of nano-Al_2_O_3_. However, nano-Al_2_O_3_ aggregated to large particles, with MHD being more than 1000 nm. Existing data suggested that nanoparticles >50 nm experience difficulty passing through the bacteria cell wall [66]. Dalai *et al.* also reported an Al uptake of only 2% at 1.0 mg/L of nano-Al_2_O_3_ into *S. obliquus* [21]. Thus, nano-Al_2_O_3_-copper complexes experience difficulty in penetrating the cell membrane. Moreover, the presence of nano-Al_2_O_3_ did not change cell membrane damage, suggesting that the possibility of copper entering the cells through the membrane was unaffected. Thus, the decrease in copper accumulation may be attributed to the decrease in the available copper in the medium as a result of copper adsorption onto nano-Al_2_O_3_. Some studies on the effect of TiO_2_ on metal toxicity have reported similar results [14,19].

## 4. Conclusions

Results of the current study showed that the existence of nano-Al_2_O_3_ decreased the toxicity of copper in the aquatic environment. The effect of nano-Al_2_O_3_ on copper toxicity mainly reduced copper accumulation in algae but did not change the toxic mechanism of copper. Low copper accumulation can be attributed to the adsorption of copper onto nano-Al_2_O_3_ and the decrease of available copper in the water. According to the literature, nano-Al_2_O_3_ had no effect on the toxicity of some other metals, such as Cr(VI). However, the toxicity of copper was reduced in the current study. This may be attributed to the fact that copper is an essential element for many organisms and the biochemical processes of copper taken up by cells is different from those of other metals. Whether the results in this paper could be extended to other essential metal elements should be further researched. Understanding these phenomena would be beneficial in the effective ecological risk assessment and management of nanomaterials and heavy metals.

## Figures and Tables

**Table 1:** Composition of the modified BG11 medium.

Component	Concentration (mg/L)	Component	Concentration (mg/L)
NaNO_3_	1500	K_2_HPO_4_	40
CaCl_2_·2H_2_O	36	MgSO_4_·7H_2_O	75
C_6_H_10_FeNO_8_	6	Na_2_CO_3_	20
C_6_H_8_O_7_	6		

**Table 2:** Concentrations of copper and nano-Al_2_O_3_ in the exposure solutions.

Concentration of Nano-Al_2_O_3_ (mg/L)	Concentration of Copper (mg/L)					
0	0	0.01	0.05	0.1	0.2	0.5
1.0	0	0.01	0.05	0.1	0.2	0.5

**Table 3:** Properties of nano-Al_2_O_3_ in the exposure solution over 72 h.

Concentration of Nano-Al_2_O_3_ (mg/L)	Concentration of Copper (mg/L)	Time (h)	MHD (nm)	PdI	Zeta Potential (mV)
1.0	0	0	439.3 ± 4.9	0.223 − 0.332	−20.5 ± 0.7
		24	540.4 ± 22.4	0.247 − 0.270	−17.7 ± 0.5
		48	1648.3 ± 122.3	0.180 − 0.222	−19.9 ± 1.2
		72	1860.0 ± 32.7	0.267 − 0.338	−20.6 ± 1.8
	0.05	0	327.1 ± 7.5	0.224 − 0.255	−19.8 ± 1.1
		24	501.3 ± 6.8	0.226 − 0.245	−18.6 ± 1.2
		48	1478.2 ± 120.6	0.322 − 0.376	−17.7 ± 0.7
		72	1720.1 ± 74.9	0.326 − 0.331	−20.8 ± 2.2
	0.5	0	336.5 ± 4.1	0.210 − 0.270	−20.1 ± 2.3
		24	338.9 ± 4.5	0.275 − 0.336	−19.4 ± 1.8
		48	1530.3 ± 161.7	0.241 − 0.250	−18.8 ± 0.7
		72	1781.4 ± 97.5	0.242 − 0.367	−20.6 ± 1.4

Notes: Data are mean ± SD (PdI are expressed as ranges, *n* = 3). MHD = mean hydrodynamic diameter. PdI = poly dispersion index.

**Table 4:** Growth inhibition (%) during exposure and copper accumulation in *S. obliquus* exposed to copper and copper/nano-Al_2_O_3_ systems after 72 h.

Concentration of Nano-Al_2_O_3_ (mg/L)	Concentration of Copper (mg/L)	24 (h)	48 (h)	72 (h)	Accumulated Copper (ng/mg)
0	0	0	0	0	35.47 ± 0.37
	0.01	−13.67	−0.96	10.05	40.83 ± 0.42
	0.05	−4.02	0.97	10.96	59.54 ± 0.01
	0.1	−6.23	7.23	17.35	81.67 ± 1.33
	0.2	−1.61	13.98	23.29	159.83 ± 2.02
	0.5	−2.41	23.63	33.33	596.04 ± 8.36
1.0	0	−16.88	−0.48	0.91	40.21 ± 0.21
	0.01	−16.87	−2.89	0.01	37.42 ± 0.01
	0.05	−9.64	4.34	10.95	55.68 ± 1.39
	0.1	−7.48	5.15	10.50	81.29 ± 2.16
	0.2	−9.64	5.79	10.96	138.62 ± 1.58
	0.5	−6.43	6.27	23.29	458.21 ± 11.09

Note: Accumulated copper is expressed as mean ± SD (*n* = 3).
